# The perpetual evidence-practice gap: addressing ongoing barriers to chronic pain management in primary care in three steps

**DOI:** 10.3389/fpain.2024.1376462

**Published:** 2024-10-08

**Authors:** Laura Ellen Ashcraft, Megan E. Hamm, Serwaa S. Omowale, Valerie Hruschak, Elizabeth Miller, Shaun M. Eack, Jessica S. Merlin

**Affiliations:** ^1^ Department of Biostatistics, Epidemiology, and Informatics, Perelman School of Medicine, University of Pennsylvania, Philadelphia, PA, United States; ^2^ Center for Health Equity Research and Promotion, Corporal Crescenz VA Medical Center, Philadelphia, PA, United States; ^3^ Qualitative, Evaluation and Stakeholder Engagement Research Services, Center for Research on Health Care, University of Pittsburgh, Pittsburgh, PA, United States; ^4^ Department of Management, Policy, and Community Health, The University of Texas Health Science Center at Houston, Houston, TX, United States; ^5^ Department of Anesthesiology Perioperative and Pain Medicine, Brigham and Women’s Hospital, Boston, MA, United States; ^6^ Department of Pediatrics, University of Pittsburgh School of Medicine, Children’s Hospital of Pittsburgh, Pittsburgh, PA, United States; ^7^ School of Social Work, University of Pittsburgh, Pittsburgh, PA, United States; ^8^ Department of Psychiatry, School of Medicine, University of Pittsburgh, Pittsburgh, PA, United States; ^9^ Division of General Internal Medicine, CHAllenges in Managing and Preventing Pain (CHAMPP) Clinical Research Center, University of Pittsburgh, Pittsburgh, PA, United States

**Keywords:** primary care, chronic pain, implementation science, qualitative research, methods

## Abstract

**Background:**

Most management of chronic pain, a serious illness affecting the physical and psychological wellbeing of millions, occurs in primary care settings. Primary care practitioners (PCPs) attempt to provide evidence-based practices to treat chronic pain. However, there continues to be a gap between the care people receive and the evidence. The objectives for this study were to (1) explore determinants of evidence-based chronic pain management and (2) develop a novel approach to using implementation science to address the evidence-practice gap.

**Method:**

A convenience sample of twenty-one Pennsylvania PCPs participated in one-time semi-structured telephone interviews. Interviews were transcribed verbatim and both deductive and inductive approaches were used during analysis. We used the Consolidated Framework for Implementation Research (CFIR) and the Expert Recommendations for Implementing Change (ERIC) to inform our analysis and findings.

**Results:**

We identified determinants of evidence-based chronic pain management across the CFIR domains of Intervention Characteristics, Characteristics of Individuals, and the Outer Setting and reported implementation strategies. Based on identified themes, we developed a three-step process to support the ongoing and pragmatic implementation of evidence-based chronic pain management in primary care settings.

**Conclusions:**

Previous efforts exist to integrate implementation science into chronic pain management; yet a gap persists. Implementation approaches should prioritize the needs of people living with chronic pain and their families. Further, future approaches or strategies used should build on the current three-step model to include the fourth step of tailoring existing implementation strategies to the specific needs of chronic pain in the clinical context.

## Introduction

Chronic pain is a common, serious illness often accompanied by psychological comorbidities including anxiety and depression (1, 2), decreased quality of life (1), reduced participation in the workforce (2), and strain on interpersonal relationships (2). It affects about 55 million people in the United States (3) and costs $635 billion annually in medical treatment and lost productivity (4). The consequences of chronic pain may be further complicated by environmental factors such as stress and lack of social support (5).

Evidence-based practices (EBPs) to treat chronic pain include pharmacologic therapies [e.g., antidepressants (6), amitriptyline, etc.], non-pharmacologic approaches such as physical therapy (7–9) and cognitive behavioral therapy (CBT) (10–12), and behavioral approaches such as self-management and cognitive behavioral therapy, yoga, and physical therapy (13). However, patients often do not receive evidence-based chronic pain management (14–16). A prime example is the treatment for chronic low back pain. A recent systematic review reported that chronic low back pain prevalence rises linearly from the third decade of life on with rates of 4.2% in individuals between 24 and 39 years old and 19.6% in those ages 20 and 59 (17), with an estimated 2.06 million cases per year (18). A 2018 series published by *The Lancet* examining current and best practices for treatment showed that low back pain results in 2.7 million emergency department visits each year, despite best practices for managing chronic pain in primary care and avoiding emergency care (19). Further, surgical procedures are often used as a strategy to manage chronic low back pain, which again contradicts guidance advising the reliance on noninvasive treatment strategies (19). One study found that only 12% of people with chronic low back pain have received psychological support and only 8.4% received CBT (19).

Primary care practitioners (PCPs; i.e., physicians, nurse practitioners, and physician assistants) (20) are often the first line of care for people living with chronic pain (20) and patients often do not receive chronic pain EBPs (14–16). In part, this is due to complex referral systems and insurance coverage (21) and a lack of pain specialists (4, 22). Therefore, understanding and integrating PCP perceptions of barriers to delivering these evidence-based therapies is a critically important next step toward improving implementation. Implementation science (IS) seeks to understand and close the gap between EBPs and clinical care (23) and may help to ensure that people living with chronic pain receive evidence-base management. The primary objectives for this study were to investigate PCP perceptions of barriers to evidence-based chronic pain management implementation, and PCP strategies (or facilitators) for addressing these barriers. The secondary objective was to develop a novel approach to using implementation science to address the evidence-practice gap in chronic pain management in primary care settings.

## Method

We selected a qualitative approach to answer our research question. Qualitative data collection and analysis support both positivist and constructionist epistemological perspectives (24). We used qualitative data collection (i.e., interviews) and analyzed data using exploratory or content-driven analysis which answers the question, “what do x people think about y?” (24). We conducted one-time semi-structured telephone interviews with PCPs between May 2019 and August 2019. The COREQ (COnsolidated criteria for REporting Qualitative research) Checklist was used and can be found in the Supplementary File S2.

### Participants and participant recruitment

Clinicians were eligible for participation if they (1) were a physician, physician assistant, or nurse practitioner; (2) practiced in an outpatient setting in Pennsylvania; and (3) treated adults. We targeted these groups as we wanted solicit insights from PCPs who are responsible for using evidence-based approaches to manage chronic pain with their patients. We limited interviews to clinicians in Pennsylvania to capture chronic pain management approaches across a diverse set of geographic areas, yet also within the same policy system (i.e., Medicaid reimbursement and prescription reporting via the PA Prescription Drug Monitoring Program). We purposively sampled for clinicians who self-reported working in either academic or non-academic settings (distinct from teaching responsibilities) to incorporate both perspectives. We used a convenience sampling approach primarily focused in Western and Central Pennsylvania agnostic to health system or affiliation with participants representing several health systems. PCPs were recruited via email and at meetings (i.e., staff meetings and in-person trainings). As such, we were unable to calculate a response rate due to the scale and broadcast nature of our recruitment strategy.

### Development of the interview guide

We developed an interview guide (see Supplementary File S1) based on the chronic pain literature and factors influencing implementation from Diffusion of Innovations Theory (25) as further operationalized by the Consolidated Framework for Implementation Research (CFIR) (26). We used the CFIR interview guide template to draft an initial interview guide and tailored it based on barriers identified in the literature and the experiences of our investigator team. For example, we solicited information on what makes it difficult to treat chronic pain with follow-up prompts focused on individual (e.g., substance abuse, depression), group (e.g., organizational difficulties), and system factors (e.g., insurance coverage).

We then pilot tested the interview guide with two academic PCPs who provided extensive feedback, and modified the original guide based on that feedback. The resulting guide contained questions focused on general chronic pain management, agnostic to the type of chronic pain treatment (i.e., pharmacologic vs. non-pharmacologic). Participants were asked to think of the types of chronic pain they commonly see in their practice and use that as a reference point for other questions. Given that evidence-based approaches to chronic pain management rather than specific pain are more similar across types and locations, we chose to focus on chronic pain generally rather than a specific pain type (e.g., neuropathic) or location (e.g., low back). Participants were then specifically prompted on the role of co-morbidities, insurance, and organizational structures.

### Data collection and transcription

The PI (LEA) conducted all interviews with iterative feedback from the research team (MEH, EM, SME, and JSM) with no field notes generated. The PI (LEA), who identifies as female, was a PhD candidate with a Master of Social Work degree, and five years of experience conducting qualitative data collection and analysis at the time of data collection. The PI introduced themselves to participants as a PhD candidate and social worker. The PI described the purpose of the study and their interest in learning more about how PCPs manage chronic pain, the challenges to that management, and ways they overcome those strategies. The PI emphasized the lived experience and expertise of each participant.

The interviews were conducted in private office with the door closed with only the PI (LEA) physically present and the participant on the phone. Participants were entered into a sweepstakes to win a $100 Amazon gift card as compensation. Interviews were audio recorded and transcribed verbatim and were continued until thematic saturation was reached and no new themes emerged. Transcripts were not returned to participants for review.

### Data coding/codebook formation and data analysis

We used an inductive approach codebook development and coding to conduct an applied thematic analysis. First, the PI developed initial codes based on the first five interviews with additional codes added as new themes emerged. Two investigators (LEA and SSO) finalized the codebook by reviewing all transcripts in duplicate until a finalized codebook was agreed upon. The PI then coded all transcripts using NVivo 12 (QSR International). Once coding was complete, the PI developed an initial set of themes from the content of the interviews.

Following this initial inductive approach, we conducted a second deductive analysis. Implementation science examines *how* evidence-based practices are put into use (23) (p5). The Consolidated Framework for Implementation Research (CFIR) is a robust meta-theoretical framework often used in implementation science which helps to identify and understand barriers to utilizing evidence-based practices (26). The Expert Recommendations for Implementing Change (ERIC) taxonomy builds on the CFIR to describe strategies (or facilitators) for overcoming identified barriers (27). For example, themes related to beneficial perceptions of treatment were identified as fitting into the relative advantage portion of the CFIR framework. Themes related to any CFIR or ERIC elements are presented in Table 1. Using this deductive approach, we mapped determinants of evidence-based chronic pain management (e.g., what makes it difficult?) identified by PCPs and mapped these to CFIR domains and constructs and mapped strategies described by PCPs to utilize evidence-based approaches to understand *how* PCPs navigate their complex environment to ERIC implementation strategies with feedback from the co-authors, but not participants.

**Table 1:** Example of steps 1–3 of leveraging IS to improve care for people living with chronic pain.

Step 1: Barriers to evidence-based chronic pain management	Examples from Steps 1 & 2	Step 3. Align Steps 1 & 2 with existing implementation frameworks	
		CFIR domain & construct	ERIC implementation strategy
Patient-level barriers			
Patient comorbidities	• Prescription side effects, drug interactions		
Patient perceptions of one treatment option over another	• Rapport building between patients and PCPs • Realistic expectations of results of chronic pain treatment	CFIR domain: intervention characteristics CFIR construct: relative advantage	Intervene with patients to enhance uptake and adherence
Practitioner-level barriers			
Lack of prior experience in measuring, diagnosing, and treating chronic pain	• Navigating non-adherence, rapport building, reliance on experience in treatment	CFIR domain: characteristics of individuals CFIR construct: self-efficacy	
Participants’ varied perceptions of external policies that increase oversight of opioid prescribing	• Fear of overprescribing pain medication	CFIR domain: outer setting cfir construct: external policy & incentives	
System-level barriers			
Lack of access to evidence-based chronic pain treatment	• On-site psychological treatment for chronic pain such as chronic pain support groups	CFIR domain: outer setting CFIR construct: patient needs & resources	Promote adaptability
	• Co-located chronic pain treatment and primary care, especially in rural areas		Change Service Sites
Financial concerns related to lack of payment for treatment	• Use of YouTube videos or other at-home exercises	CFIR domain: outer setting CFIR construct: external policy & incentives	Develop disincentives
	• Save up for co-pays		Use other payment schemes
	• Use payment program		
Lack of support to address social determinants of health that impact chronic pain	• Transportation barriers; cultural definitions of pain; systemic racism; income inequality	CFIR domain: outer setting CFIR construct: cosmopolitanism	

The University of Pittsburgh Human Research Protection Office approved this study (STUDY19010045). All participants provided verbal informed consent prior to participation.

### Three-step approach to integrating implementation science

**Figure 1:**
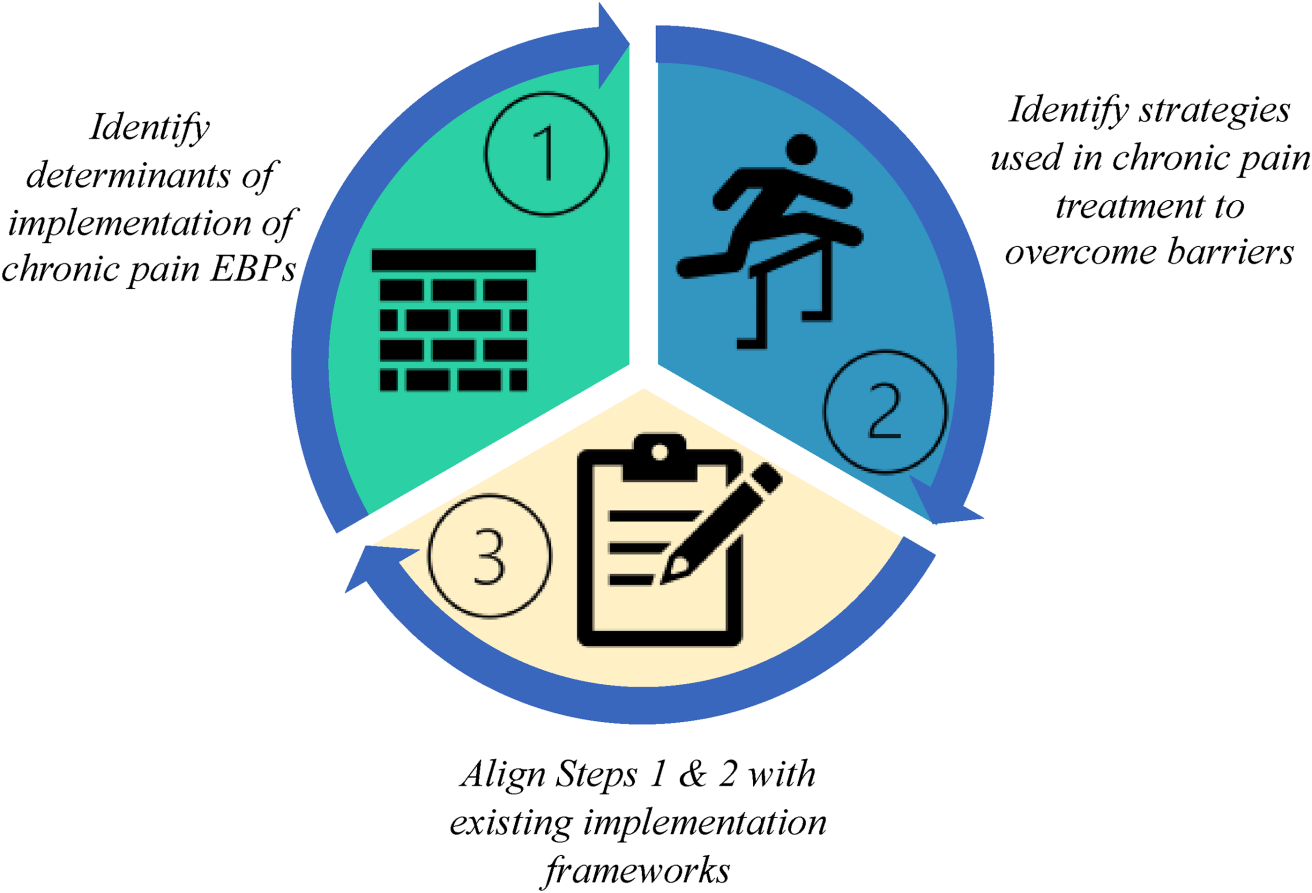
Leveraging implementation science to improve care for people living with chronic pain.

Based on our experience, we developed a three-step approach to identifying the evidence-practice gap through the process of analyzing the interview data and mapping it to existing implementation science theories and frameworks (Figure 1). The steps are as follows: Step 1: Identify determinants of implementation of chronic pain EBPs, Step 2: Identify strategies used by PCPs providing chronic pain treatment to overcome barriers, and Step 3: Align Steps 1 & 2 with existing implementation frameworks which leverages existing knowledge across the field of IS. In the current study, we tested the feasibility of using this approach for chronic pain management in primary care settings by conducting a qualitative study of PCPs.

## Results

We interviewed 21 PCPs (see Table 2). The mean age was 51 (*SD* = 10.8). Half of participants (*n* = 11) identified as male, and most participants (*n* = 17) identified as white, with three participants identifying as Asian. The average number of years post-training was 21 (range 3–35); participants spent an average of 3.3 days (range 0.5–5) in the clinic per week. Participants were mostly physicians with 61% (*n* = 13) being Medical Doctors (MDs) and 28% (*n* = 6) as Doctors of Osteopathy (DOs). The remaining two participants were advanced practice providers (one Physician Assistant and one Nurse Practitioner). Half of the participants worked in an academic clinic (*n* = 10), and most (*n* = 18) were involved in some form of teaching (e.g., supervising medical residents). Interviews were an average of 21 min 14 s (range: 15:58–25:05). No participants refused participation nor dropped out for the duration of the interviews.

**Table 2:** Participant demographics (*n* = 21).

	*n* (%)	Mean (SD)
Profession		
MD	13 (61.9)	
DO	6 (28.6)	
PA-C	1 (4.6)	
CNP	1 (4.6)	
Gender (Male)	11 (52.4)	
Age^a^		51.0 (10.8)
Race (White)^a^	17 (81.0)	
Years Post Training		20.9 (9.8)
Clinic days per week		3.3 (1.5)
Academic	10 (47.6)	
Teaching	18 (85.7)	

^a^ *n* = 20, one participant declined to provide their age and race.

Results from Steps 1-3 are as follows: Step 1. Identify determinants of clinician's implementation of chronic pain EBPs. Participants reported barriers to implementing chronic pain EBPs in primary care settings across patient, provider, and system levels. A major issue cited was access to non-pharmacologic treatment such as cognitive behavioral therapy and physical therapy. One participant described these barriers in this way:

“I would say insurance coverage is always an issue, especially when people are looking for things like aqua therapy and acupuncture and things that may not be covered well by insurance. Also, physical therapy, although it's “covered,” a lot of times, the out-of-pocket expense, though, is still too high for patients to afford. So, some things are covered but not to the point of being able to be affordable.” Participant # 9; Physician, MD

Step 2. Identify strategies clinicians used in providing chronic pain treatment to overcome barriers. PCPs reported strategies they use to overcome these barriers such as using existing resources, rapport with patients, and understanding of the health system. For the barrier of access, some participants described developing in-house psychological treatment for people living with chronic pain. A participant described this approach used by their clinic to address access to psychological treatment for chronic pain management:

“So often times we'll talk about– we actually have a local group that meets with a counselor or a psychologist too. He just talks about chronic pain and how do you live with it and what do you do personally to manage the pain. Because we know that oftentimes we can't completely eliminate or most of the time we can't eliminate pain we could just make it bearable. So, starting with offering that group.” Participant #11; Physician, MD

Step 3. Align Steps 1 & 2 with existing implementation frameworks. The PI (LEA) reviewed the barriers and strategies identified from our inductive coding of interview transcripts and mapped them onto existing CFIR domains and constructs and ERIC implementation strategies. These selections were then reviewed by co-investigators (LEA, MEH, SME, and JSM) for acceptability from a qualitative (MEH) and chronic pain (JSM) perspective. We did this by reviewing the definitions of CFIR domains and constructs and implementation strategies with the identified themes from the data and matched definitions. For example, the barrier to access aligned with the CFIR Outer Setting domain and construct of Patient Needs and Resources because the PCP recognized the access need of patients living with chronic pain. The strategy to overcome aligned with the ERIC implementation strategy of Change Service Site because they moved treatment to a setting easily accessible by patients (i.e., in-house treatment). In the interviews, participants did not suggest any strategies to address the patient-level barrier of co-morbidities nor practitioner-level barriers. Table 1 has a list of themes that arose through our data collection and analysis and their alignment with existing implementation frameworks.

## Discussion

Our results, while not generalizable, align with previous research within chronic pain management in primary care settings: the evidence-practice gap persists, particularly for implementation of non-pharmacologic approaches, despite ongoing efforts to integrate IS principles into chronic pain management (12, 28–34) and providers using creative approaches to ensure their patients receive the care they need. The importance of the therapeutic relationship between the patient and PCPs is well-known. The ongoing patient-practitioner relationship often helps to frame and interpret difficult conversations, including those about chronic pain (35). Conversations about chronic pain management can be difficult as PCPs may struggle to believe patients' reported pain (36) and patients may struggle to feel trusted in their own experience (35).

However challenging, participants in our study expressed a degree of hope and at times desire to push through the hard conversations to address a patient's pain or possible substance dependency. Chronic pain management still exists within the shadow of the opioid epidemic. The consequences of opioid use (and misuse) continue to be pervasive in national dialogue, continuing education, and funding—especially given the uptick in opioid-related deaths during the COVID-19 pandemic (37, 38). Again, given these challenges, across the board, practitioners in our study expressed the desire to navigate the complex challenges of treating chronic pain with their patients despite these contextual barriers. Participants demonstrated an immense desire to work with patients to navigate complex systems and manage their chronic pain. Unexpectedly, PCPs in this study were open to identifying and using non-opiate treatments to help manage their patient's chronic pain, even as these treatment options were typically harder to access. Participants showed surprising resourcefulness in overcoming known barriers to get patients the help they need, such as hosting chronic pain support groups in the primary care clinic. These strategies create an innovative foundation for future testing and implementation in more diverse clinical settings.

We developed a three-step approach as one way to interpret limited samples of provider experiences specifically within chronic pain to begin to address implementation gaps within a given clinical context. Our approach attempts to leverage the field of implementation science by providing a way for researchers and practitioners alike to address this gap by taking a strengths-based approach to the experience of PCPs and how they continue to find innovative ways to navigate complex chronic pain management. We then used the field of implementation science to complement and build on the front-line knowledge of barriers to treatment and the navigation of those barriers. Alignment of current barriers and strategies with the larger field of implementation science may provide avenues for additional strategy discovery or approaches used in other settings which can promote the ongoing evolution of navigating the complex barriers to treating chronic pain.

**Figure 2:**
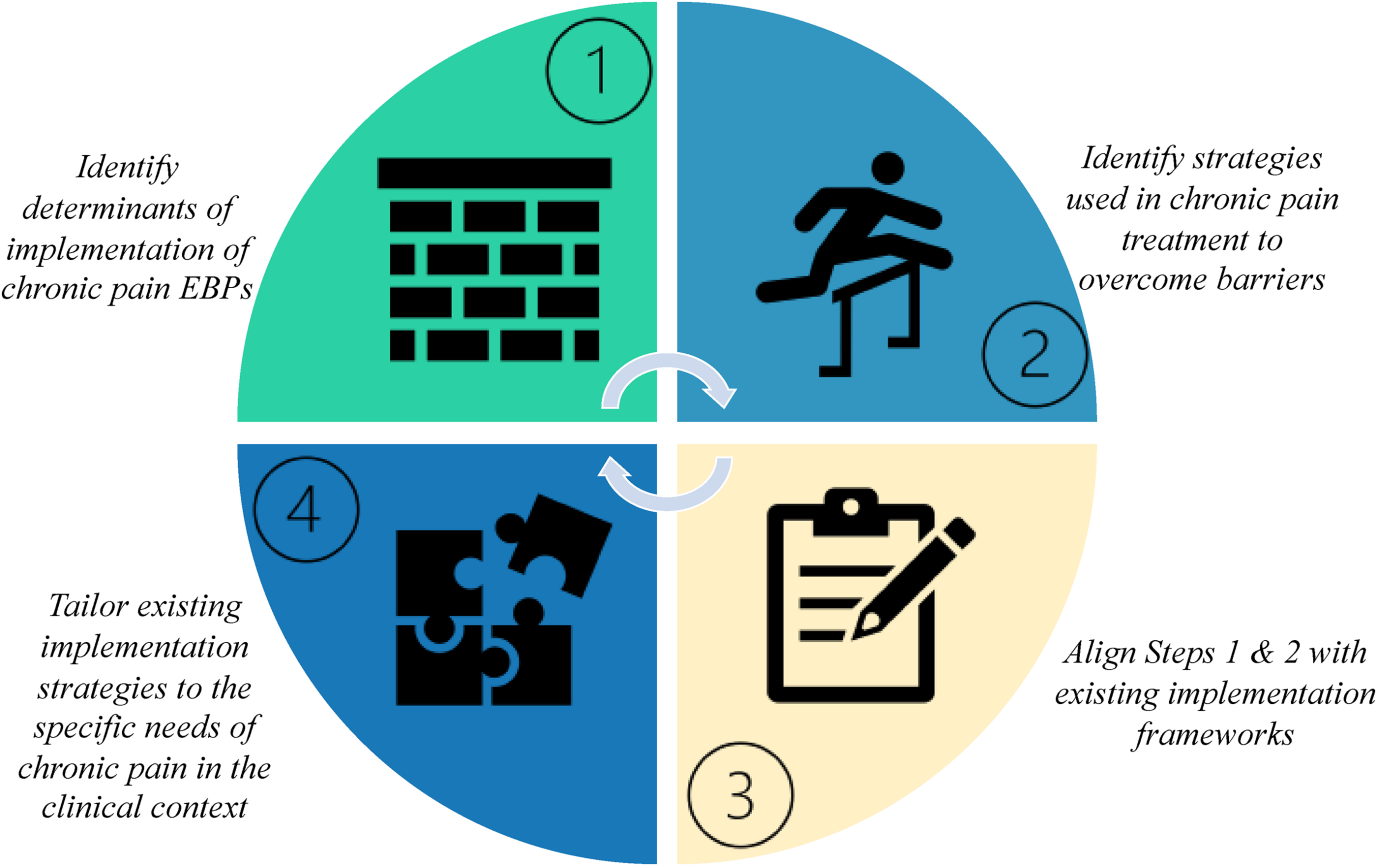
Putting implementation science into action to improve care for people living with chronic pain.

Clinicians, scholars, and quality improvement evaluators can leverage existing knowledge by aligning existing barriers and strategies with the larger literature. Future practice and research should focus on building on the existing model to include Step 4 tailor existing implementation strategies to the specific needs of chronic pain in the clinical context, perhaps by formalizing changes in service location (per our earlier example) and expanding it to include virtual support groups and teletherapy (see Figure 2). Based on our findings, approaches should be patient-centered and solicit guidance from people living with chronic pain and their families and support systems. Strategies to overcome barriers and the interventions themselves should be adaptable and tailored to the given clinical context, type of chronic pain, and patient preferences. Important considerations include the degree to which the policy setting may impact PCPs ability to navigate barriers to evidence-based chronic pain management. Some contextual factors may include the degree to which mental health services reimbursed at the same or similar rates to physical health services (mental health parity) or the impact of the expansion of Medicaid influence the proportion of people with insurance coverage.

### Limitations

The current study has several limitations. First, these findings represent a limited convenience sample and the reported experiences of participants in Pennsylvania—which are not generalizable. Most participants self-identified as white. Additionally, we did not solicit feedback on the identified implementation strategies from participants and instead relied on the qualitative and clinical experience of the investigative team. Future research should solicit the experiences of non-white PCPs to assess whether their approaches to managing patients with chronic pain differ from those of their white counterparts. These data were collected before the COVID-19 pandemic and therefore we may be missing some now critical updates to the way in which chronic pain management occurs. The potential for social desirability bias is also a concern. The PI developed rapport with each participant throughout the recruitment, screening, and interview process by highlighting the lived experience and expertise of the participants as PCPs.

### Conclusion

The gap between evidence-based chronic pain management and care for chronic pain persists even with ongoing efforts to integrate IS. We developed a three-step process to show barriers that PCPs continue to face and strategies to overcome with the goal to integrate local information and IS knowledge to improve patient outcomes.

## Data Availability

De-identified data supporting the conclusions of this article will be made available upon reasonable request to the authors.

## Supplementary material

Supplementary File S1 Interview Guide.

Supplementary File S2 COREQ Checklist.
