# Supplementary material for: The perpetual evidence-practice gap: addressing ongoing barriers to chronic pain management in primary care in three steps
Source: Front Pain Res (Lausanne). 2024 Oct 8;5:1376462. doi: 10.3389/fpain.2024.1376462 (PMC11493740; doi:10.3389/fpain.2024.1376462)
Supplement: Supplementary File S1 — Interview Guide. [file Datasheet1.docx]

Appendix 1: Interview Guide

Chronic pain is an incredibly broad topic. When thinking generally about chronic pain, tell me about your experience in treating patients with chronic pain.

- *If necessary:* What type of chronic pain do you see most often with your patients?
  - For the rest of our interview, think about this type of chronic pain.

What treatment or treatments do you usually try first?

- What makes you choose that treatment?—Emphasize why/rationale
- What do you try next if that doesn’t work?
  - What makes you choose that treatment?

There are many evidence-based ways to treat chronic pain from pharmacological to non-pharmacological approaches, what are some strategies that you’re familiar with?

What are some things that make it difficult to use evidence-based chronic pain treatments with your patients?

- Prompts: Comorbidities: depression, substance use, etc., Insurance/Financing, Organizational difficulties
- Avoid saying barriers!

How do you know about the evidence-base for these strategies?

- Prompts: Content of webinar/conferences/articles

We’ve talked about a lot, what else should we know about chronic pain treatment evidence and difficulties using these treatments?

Demographic Questions:

- What is your profession?
- What licenses do you hold?
- What sub-specialties do you have, if any?
- What is your title?
- How many years have you practiced since you finished your training?
- How many days per week you work in the clinic?
- Do you engage in educating students or trainees?
- In what type of setting do you practice?
- What is your age?
- How do you identify your race and ethnicity?
- How do you identify your gender?

We are at 20 minutes. I have a few follow-up questions. Do you have a few more minutes to talk?

*Additional follow-up questions after the 20 minute mark included any missed questions or prompts and/or follow-up on any previous comments related to the research questions.*
